# Ear Ache

**Published:** 1890-03

**Authors:** 


### EAR ACHE.

---

Take five parts of camphorated chloral, thirty parts of glycerine, and ten parts of oil of sweet almonds. A piece of cotton is saturated and introduced well into the ear, and it is also rubbed behind the ear. The pain is relieved as if by magic, and if there is inflammation it often subsides quickly.—*Medical Briefs.*

